# Supplementary material for: Analytical Performance of NGS-Based Molecular Genetic Tests Used in the Diagnostic Workflow of Pheochromocytoma/Paraganglioma
Source: Cancers (Basel). 2021 Aug 22;13(16):4219. doi: 10.3390/cancers13164219 (PMC8392134; doi:10.3390/cancers13164219)
Supplement: Supplementary file 1 [file cancers-13-04219-s001.zip › cancers-1351335-supplementary.pdf]

Supplementary Table S1: Sequencing platforms of WES.

	Illumina HiSeq 2000		Complete Genomics
**Library preparation**	**SureSelect Agilent 51M**	**Illumina Nextera Rapid Capture Exome v1.2**	**BGI 59Mb exome kit**
**Sequencing instrument**	HiSeq 2000	HiSeq 2000	Complete Genomics
**Primary analysis**	Illumina base calling Software 1.7	Illumina base calling software 1.7	Complete genomics RTA 1.7 software
**Aligment**	Burrows-Wheeler Aligner (BWA)	Burrows-Wheeler Aligner (BWA)	Teramap
**Annotation**	GATK workflow (Samtools, Annovar)	GATK workflow (Samtools, Annovar)	GATK workflow (Samtools, Annovar)

Supplementary Table S2: Distribution of sequencing reads obtained by different WES platforms

Mean values	**SureSelect Agilent 51M**	**BGI 59Mb exome kit**	**Illumina Nextera Rapid Capture Exome v1.2**
reads aligned to the human genome (%)	98,68	100	99,88
unique reads (%)	61,6	56,7	53,5
bases corresponding to targeted sequences (%)	51,6	58,9	50,38
target bases with no coverage (%)	1,32	0	0,13

Supplementary Table S3: Mean depth of coverage of PPGL genes obtained during WES.

Gene	SureSelect Agilent 51M, read numbers (range)	BGI 59Mb exome kit read numbers (range)	Illumina Nextera Rapid Capture Exome v1.2 read numbers (range))
EGLN1	112,7 (94,4–140,5)	58,5 (49,1–65,4)	121,4 (97,3–146,7)
EPAS1	110,5 (101–123,5)	77,4 (48,7–89,7)	149,0 (115,7–192,6)
FH	82,6 (73,2–103,2)	58,0 (50,2–68,8)	132,4 (106,2–155,1)
GOT2	77,0 (71,6–85,5)	95,0 (77,4–105,6)	50,6 (40,6–61,3)
KIF1B	141,8 (132,4–155,3)	103,5 (84–116,6)	148,9 (120–184,2)
MAX	105,3 (92–126,7)	80,8 (66,5–87,4)	147,8 (120–184,2)
MDH2	105,2 (96,5–117,7)	90,7 (98,7–103,8)	58,8 (46,2–71,7)
MEN1	122,5 (112–143,5)	95,7 (68,9–109,2)	110,4 (94,2–143,7)
NF1	84,5 (78–100)	109,4 (95,2–124,1)	129,6 (100–158,2)
RET	97,2 (87,6–107,8)	68,7 (48,2–75,7)	132,3 (106,5–173,6)
SDHA	11,7 (10,5–14,4)	66,3 (59,6–71,8)	175,9 (147,8–217,5)
SDHAF2	118,8 (112,5–128,3)	90,2 (81,1–108,4)	166,9 (145–205,7)
SDHB	98,8 (85,3–112,8)	52,9 (41,2–62,6)	158,1 (117,7–199,2)
SDHC	34,4 (28,1–42,9)	114,8 (90,8–127,3)	186,9 (140,3–227)
SDHD	19,4 (14,7–23,6)	118,2 (102,3–130,4)	138,3 (101,5–172,3)
SLC25A11	141,4 (126,2–165,3)	147,6 (101,5–169,2)	62,8 (51,8–81,8)
TMEM127	84,8 (70,9–90,7)	47,0 (31,3–53,1)	112,8 (86,8–144)
VHL	126,3 (116,9–143,9)	59,5 (49–67,9)	126,2 (105–164,2)
